# Disentangling Genetic Variation for Resistance and Endurance to Scuticociliatosis in Turbot Using Pedigree and Genomic Information

**DOI:** 10.3389/fgene.2019.00539

**Published:** 2019-06-07

**Authors:** María Saura, María J. Carabaño, Almudena Fernández, Santiago Cabaleiro, Andrea B. Doeschl-Wilson, Osvaldo Anacleto, Francesco Maroso, Adrián Millán, Miguel Hermida, Carlos Fernández, Paulino Martínez, Beatriz Villanueva

**Affiliations:** ^1^Departamento de Mejora Genética Animal, INIA, Madrid, Spain; ^2^CETGA, Cluster de la Acuicultura de Galicia, Aguiño-Ribeira, Spain; ^3^Genetics and Genomics, The Roslin Institute and R(D)SVS, The University of Edinburgh, Roslin, United Kingdom; ^4^Geneaqua, Lugo, Spain; ^5^Departamento de Xenética, Universidade de Santiago de Compostela, Lugo, Spain

**Keywords:** aquaculture, disease, resilience, resistance, endurance, scuticociliatosis, turbot

## Abstract

Selective breeding for improving host responses to infectious pathogens is a promising option for disease control. In fact, disease resilience, the ability of a host to survive or cope with infectious challenge, has become a highly desirable breeding goal. However, resilience is a complex trait composed of two different host defence mechanisms, namely resistance (the ability of a host to avoid becoming infected or diseased) and endurance (the ability of an infected host to survive the infection). While both could be targeted for genetic improvement, it is currently unknown how they contribute to survival, as reliable estimates of genetic parameters for both traits obtained simultaneously are scarce. A difficulty lies in obtaining endurance phenotypes for genetic analyses. In this study, we present the results from an innovative challenge test carried out in turbot whose design allowed disentangling the genetic basis of resistance and endurance to *Philasterides dicentrarchi*, a parasite causing scuticociliatosis that leads to substantial economic losses in the aquaculture industry. A noticeable characteristic of the parasite is that it causes visual signs that can be used for disentangling resistance and endurance. Our results showed the existence of genetic variation for both traits (heritability = 0.26 and 0.12 for resistance and endurance, respectively) and for the composite trait resilience (heritability = 0.15). The genetic correlation between resistance and resilience was very high (0.90) indicating that both are at a large extent the same trait, but no significant genetic correlation was found between resistance and endurance. A total of 18,125 SNPs obtained from 2b-RAD sequencing enabled genome-wide association analyses for detecting QTLs controlling the three traits. A candidate QTL region on linkage group 19 that explains 33% of the additive genetic variance was identified for resilience. The region contains relevant genes related to immune response and defence mechanisms. Although no significant associations were found for resistance, the pattern of association was the same as for resilience. For endurance, one significant association was found on linkage group 2. The accuracy of genomic breeding values was also explored for resilience, showing that it increased by 12% when compared with the accuracy of pedigree-based breeding values. To our knowledge, this is the first study in turbot disentangling the genetic basis of resistance and endurance to scuticociliatosis.

## Introduction

Infectious diseases represent a major threat to farmed animal populations. In addition to their impact on health and welfare of the affected animals and the associated production losses, they can also have implications for food security and human health (Wiethoelter et al., 2015). In recent years, genetic disease control strategies through artificial selection for enhancing host response to infectious pathogens have received increasing interest. In particular, disease resilience, the ability of a host to survive or cope with infectious challenge, has become a highly desirable breeding goal (Bisset and Morris, 1996; Ødegård et al., 2011a; Hermesch, 2014; Colditz and Hine, 2016; Gjedrem and Rye, 2016; Houston, 2017). However, resilience to infections is a complex trait composed of different types of response mechanisms (Bisset and Morris, 1996; Doeschl-Wilson and Lough, 2014; Colditz and Hine, 2016). For diseases with potential lethal outcome, two host traits in particular affect survival. These are resistance, here defined as the ability of a host to avoid becoming infected or diseased when exposed to infectious material, and endurance, here defined as the ability of the host, once infected or diseased, to survive the infection (Ødegård et al., 2011b). Note that endurance is closely related to tolerance, which refers to the ability of an infected host to reduce the fitness consequences of infection (Roy and Kirchner, 2000). However, to date, the relative importance of either mechanism (resistance and endurance) to survival is unknown. Disentangling both components is critical in genetic improvement programmes because they could be antagonistically related and also because they may have different effects on disease spread in a population; whereas individuals with high resistance likely reduce disease spread, infected individuals with high endurance may be more tolerant and transmit infections for longer.

The existence of genetic variability is a prerequisite for obtaining genetic responses through selection but very few studies have been designed to estimate the genetic basis of both resistance and endurance. In fact, most studies investigating host genetics for infectious diseases have focused on host resistance (Bishop and Woolliams, 2014), as endurance is difficult to quantify or analyse from available animal disease data (Ødegård et al., 2011b; Kause and Ødegård, 2012).

The consequences of infectious diseases on animal production seems to be most problematic in the conditions of aquaculture settings. The confinement in tanks or cages, where many individuals share a common environment, highly facilitates the transmission of infections. Indeed, the economic impact of diseases on aquaculture, entails losses of more than six billion US $ per year (World Bank, 2014). In selection programmes designed for improving resistance to disease, phenotypes are commonly obtained from challenge tests for specific pathogens. Given that challenged fish cannot be used as breeding candidates, selection is based on sib’s performance and thus, only the between-family component of the genetic variance can be exploited when using only pedigree information (Nielsen et al., 2009). A potential way to exploit the within-family component would be to apply genomic selection (Meuwissen et al., 2001) for which important benefits have been showed from computer simulations (Nielsen et al., 2009; Sonesson and Meuwissen, 2009; Villanueva et al., 2011; Lillehammer et al., 2013) and analyses of data of different aquaculture species (Ødegård et al., 2014; Palaiokostas et al., 2016, 2018a,b; Tsai et al., 2016). An alternative would be to apply marker assisted selection (MAS) if major QTLs have been identified (e.g., Houston et al., 2008; Moen et al., 2009). Given the recent history of selective breeding for most aquaculture species (Gjedrem et al., 2012), it could be expected that major genes for disease traits are still segregating in commercial populations.

Within aquaculture species, turbot (*Scophthalmus maximus*) is the main flatfish produced worldwide due to its high commercial value. This has prompted its intensive farming during the last decades and a fast development of genomic resources for the species (Martínez et al., 2016). World production of farmed turbot reached over 65,000 t in 2015 while total catches were only about 6,000 t (EUMOFA, 2018). One of the main threats for the growing turbot aquaculture sector are the pathologies caused by bacteria, viruses, and parasites (Pereiro et al., 2016). Specifically, scuticociliatosis, a disease caused by the histophagus ciliate *Philasterides dicentrarchi*, is responsible for very important economic losses not only for the turbot industry, but also for other aquaculture species such as the olive flounder (*Paralichthys olivaceus*), fine flounder (*Paralichthys adspersus*), European sea bass (*Dicentrarchus labrax*) and kelp grouper (*Epinephelus bruneus*) among others (Harikrishan et al., 2012; Folgueira et al., 2018). Although the immune mechanisms against scuticociliates are unclear (Piazzon et al., 2013), a relevant characteristic of *P. dicentrarchi* is that it causes visual conspicuous signs that can be used as proxy of the time of onset of disease, therefore allowing disentangling resistance and endurance.

In this study, we analysed data from an experiment carried out in turbot infected with *P. dicentrarchi* in order to estimate genetic parameters and identify potential QTLs for resistance, endurance and the composite trait resilience, using pedigree and high-throughput SNP data. The accuracy of genomic selection was also estimated to determine its potential.

## Materials and Methods

### Data and Trait Definitions and Measurements

Data used in this study came from an innovative transmission experiment specifically designed for disentangling the different components of the host response to infection, using scuticociliatosis in turbot as a model. The different components include not only resistance and endurance but also infectivity (i.e., the propensity of an infected individual to transmit the disease). Given that the statistical models for genomic analyses of infectivity are not fully developed yet, here we focus on the two first components (i.e., resistance and endurance). We also considered the composite of both traits; i.e., resilience. This is the trait most commonly recorded in aquaculture species to address disease resistance in fish.

*Philasterides dicentrarchi* causes systemic infection in turbot, invading internal organs such as brain, gills, liver, and intestine that generally results in the death of the host (Paramá et al., 2003). This parasite has been associated with various pathological changes including exophthalmia, colour change or depigmentation, visible lesions or abnormal swimming behaviour (Piazzon et al., 2013). Here, the appearance of visual infection signs was used as a proxy for time of onset of disease. Accordingly, the resistance phenotype was the number of days to the onset of visual signs, whereas the endurance phenotype was the number of days from the onset of visual signs to death; the resilience phenotype was then the number of days from the start of the experiment until death.

One thousand and eight hundred individuals from 44 full-sib families (29 sires and 25 dams) were challenged (either by injection or cohabitation with infected individuals, as described below) with *P. dicentrarchi* at the Cluster de Acuicultura de Galicia (CETGA, NW Spain) facilities. The CETGA’s broodstock represent a population of Atlantic origin (Maroso et al., 2018) that was founded with individuals sampled just before or at the start of the main selection programmes established in Europe (Janssen et al., 2017). Due to space limitations, the experiment was carried out in two consecutive trials although families for both trials were created at the same time. There were 900 fish and 22 families for each trial. The trials were run until no new infections or deaths were observed in most of the tanks after 1 week and lasted for 104 (trial 1) and 160 (trial 2) days. Overall mortality rate was 69% in trial 1 and 64% in trial 2. Both fish that died during the experiment and fish that survived at the end of the experiment were examined for the presence of parasites.

**Table 1:** Duration of each trial (days), number of tanks, sires and dams, shedder and recipient families and fish used in the challenge experiment.

	Trial 1	Trial 2	Total
Days	104	160	264
No. of tanks	36	36	72
No. of sires	10	13	23
No. of dams	13	13	26
No. of families	22	22	44
Shedder	4	4	8
Recipient	18	18	36
No. of individuals	900	900	1,800
Shedder	180	180	360
Recipient	720	720	1,440

The detailed experimental design is given in Anacleto et al. (2019). Briefly, fish were distributed in 72 tanks (36 in each trial). Each tank contained five shedder fish from the same family previously infected with the parasite by intraperitoneal injection. Shedder fish were inoculated with 200 μl of sterile physiological saline containing 50,000 and 56,000 ciliates for trials 1 and 2, respectively, given the lower weight of fish and the higher virulence of the isolate of *P. dicentrarchi* used in trial 1 (see below). In addition, each tank contained 20 recipient fish (originally non-infected) from four different families (five fish per family). These four families constituted a recipient family combination. In each trial, the total number of shedder families and family combinations were four and nine, respectively. Each of the four shedder families infected all nine family combinations (4 × 9 = 36 tanks) by cohabitation. Each recipient family was represented in eight tanks and in two different family combinations. A summary of numbers of fish and families used in each trial is given in Table 1.

Fish were inspected twice a day for visual signs and mortality, and dates of first detection of visual signs or death were collected. Weight of fish was recorded at the start and at the end of the challenge (or at the time of death). The average weight (g) of fish was 32.9 (SD = 9.5) and 88.5 (SD = 27.3) in trials 1 and 2, respectively. Individual fish were identified using elastomers (families) and fin clipping (individuals within a family). Fin tissue samples were collected for DNA extraction and genotyping at GeneAqua facilities.

The data analysed corresponded to the 1,440 recipient fish challenged by cohabitation. These fish belonged to 36 full-sib families (i.e., 40 fish per family) created from 23 sires and 23 dams that were unrelated. These families included 12 paternal half-sib families (11 males were mated with 1 female, 11 males with 2 females, and 1 male with 3 females) and 11 maternal half-sib families (12 females were mated with 1 male, 9 female with 2 males and 2 females with 3 males). Both trials were connected through two male and three female parents.

Genome-wide SNP data were available for 1,394 offspring (46 fish failed quality control) from the 36 full-sib families and their 46 parents. Genotypes were obtained using a 2b-RAD sequencing approach as described in Maroso et al. (2018). Briefly, after mapping to the turbot reference genome (Figueras et al., 2016) and applying quality filters (Maroso et al., 2018) an initial set of 25,511 SNPs was obtained. From them, only those present in 80% of parents and with a minimum coverage of 10× were retained. This set of SNPs was used as a reference to obtain the SNPs in the offspring. Markers showing Mendelian errors (offspring genotype being inconsistent with Mendelian transmission, given the parental genotypes), unmapped SNPs and those with MAF < 0.015 in the parent population and with extreme departures of Hardy–Weinberg equilibrium (*p* < 0.001) were removed. Also, for tags containing multiple polymorphisms only one SNP was retained. After quality control, a total of 18,125 SNPs were retained. The assembly of the turbot genome (Figueras et al., 2016) has been recently improved through a high-density genetic map (Maroso et al., 2018) and 97% of the genome has been anchored constituting 22 megascaffolds in accordance with its karyotype constitution. We followed the linkage group (LG) nomenclature of Maroso et al. (2018), where LG18 has been merged with LG8 (i.e., LG8+LG18 is now LG8) when compared to the previous version of the map (Figueras et al., 2016).

### Estimation of Genetic Parameters for Traits Associated With Host Response to Infection

#### Environmental Effects and Heritabilities

The analysis of the genetic and environmental components of resistance, endurance, and resilience was carried out using survival analysis methodology. Survival analysis is optimal to analyse time-to-event variables because it allows the use of information from incomplete records (censored data), it takes into account factors that determine the risk of the event of interest over time and relies on functions that adequately represent survival processes. Those individuals that survived to the end of the experiment were considered as censored data for resilience; those that survived without showing any visual sign were considered as censored for resistance, and those that had shown signs but survived to the end of the experiment were considered as censored for endurance.

A non-parametric Kaplan–Meier (KM) estimator of the survival function was obtained for each recipient family and for each trial in order to assess the existence of variation between family and trial groups for the three traits analysed.

Significance of systematic effects on survival was determined by likelihood ratio tests using the Cox partial likelihood of the data under the complete (with all systematic effects) and reduced models (i.e., evaluating each effect at a time) using the following model

(1) $$h(t)=h_{0}(t)\times \exp(\mathrm{Xb}),$$

where *h*(*t*) is the risk of onset of visual signs (resistance) or death (resilience and endurance) at time *t*; *h*_0_(*t*) is the baseline function at time *t* (Cox model) stratified by trial, **b** is the vector of systematic effects including the weight at the start of the experiment (that ranged from 10.1 to 196.3 g, and for which ten levels were considered) and the tank (71 levels as one tank in trial 1 was discarded due to a problem with oxygen supply), and **X** is the incidence matrix relating risk at time *t* to the corresponding level of systematic effects. Note that sources of variation within tanks come from a combination of the environmental conditions of each tank, shedder family, recipient family combination and trial. Overall goodness of fit was assessed by the statistic $R_{\text{M}}^{2}$ of Maddala (1983) which is a measure of the proportion of the overall variation explained by the model.

The general model fitted to estimate the variance components of the disease traits was a proportional hazards mixed model of the form

$$h(t)=h_{0}(t)\times \exp\;(\mathrm{Xb}+\mathrm{Zu}),$$

where *h*(*t*), *h*_0_(*t*), **X** and **b** are as in (1), **u** is a vector of animal genetic effects, and **Z** is the design matrix for **u**. Animal genetic effects were assumed to be distributed as a *N* (0, **A**${\text{σ}}_{\text{u}}^{2}$), where **A** is the numerator relationship matrix for all fish included in the pedigree and ${\text{σ}}_{\text{u}}^{2}$ is the additive genetic variance. Heritability estimates were obtained both in logarithmic and original scales. The estimate in the logarithmic scale was obtained following Ducrocq and Casella (1996) as

$$h_{\log}^{2}$$

= ${\text{σ}}_{\text{u}}^{2}$/(${\text{σ}}_{\text{u}}^{2}$ + π^2^/6). The heritability in the original scale was estimated following Yazdi et al. (2002) as *h*^2^ = ${\text{σ}}_{\text{u}}^{2}$/(${\text{σ}}_{\text{u}}^{2}$+ (1/*p*)), where *p* is the proportion of uncensored data.

The previous mixed model was solved both with semi-parametric Cox and with parametric Weibull approaches. The use of the Cox model does not require specification of the distribution of the independent variable, but it has a larger computational burden and it is more affected by the data structure than Weibull models. The hypothesis of a Weibull distribution was graphically tested by regressing log (-log *S*(*t*)) against log *t*, where *S*(*t*) is the survival function. A straight line is expected if the Weibull model fits well.

The Survival Kit V.6.1 software (Mészáros et al., 2013), which follows a Bayesian approach to solve for the unknowns in the model, was used. The posterior density of the genetic variance was estimated using the Gram–Charlier approximation (Mészáros et al., 2013) and the probability of the additive genetic variance being larger than 0 was computed.

#### Genetic Correlations

Estimation of genetic correlations between the disease traits and also between these traits and growth was carried out under a standard linear model framework given the complexity and the lack of software available to estimate genetic correlations using survival analysis techniques. Standard linear models cannot handle censored data but they can easily accommodate multi-trait models and the use of genomic relationship matrices, which provide more precise information about the genetic relationships between individuals. Growth rate was measured as the difference in weight between the end (or the time of death) and the start of the experiment divided by the number of days. Pairwise correlations between resilience, resistance and endurance and between these traits and growth rate were estimated fitting bivariate animal models for each pair of traits of the following form

(2) y=Xb+Zu+e,

where **y** is the vector of phenotypic records for a pair of traits, **b** is the vector of fixed effects (weight and tank effects for both traits), **u** and **e** are vectors of random animal genetic and residual effects, respectively, and **X**, **Z** are the design matrices for the corresponding effects. Here, var(**u**) = **G_o_** ⊗ **G**, where ⊗ denotes the Kronecker product, **G**_o_ is the 2 × 2 additive genetic (co)variance matrix between the two traits and **G** is the genomic relationship matrix computed as in VanRaden (2008), and var(**e**) = **R**_o_⊗**I**, where **R**_o_ is the (co)variance matrix between residual terms for each trait and **I** is the identity matrix. Bayesian estimates for (co)variance components in **G**_o_ and **R**_o_ were obtained via Gibbs sampling using the software GIBBS1F90 (Misztal et al., 2002). The number of Gibbs samples was 110,000. The first 10,000 were discarded and then one sample every 20 was considered.

### Accuracy of Genomic Selection and Detection of QTL Regions Associated With the Traits Defining the Host Response to Infection

In order to evaluate gains in accuracy from using genomic information, estimates of genetic and genomic breeding values were obtained from standard BLUP and from GBLUP (VanRaden, 2008), respectively. Both BLUP and GBLUP were carried out with the BLUPF90 family of programmes (Misztal et al., 2002). The model assumed was as in (2) but in a univariate setting and thus, var(**u**) was **A**${\text{σ}}_{\text{u}}^{2}$ and **G**${\text{σ}}_{\text{u}}^{2}$ for BLUP and GBLUP, respectively. The accuracy of pedigree-based and genomic evaluations was estimated using a 10-fold cross-validation test. Families were randomly split into ten groups. Within each group, 90% of the fish were chosen to be part of the training set and the rest was part of the validation set. The accuracy was estimated as *r*(*y*, *ŷ*)/*h*, where *r*(*y*, *ŷ*) is the correlation between the observed and predicted phenotype, and *h* is the square root of the heritability (Legarra et al., 2008).

Mixed linear models were also fitted to perform genome wide association analyses (GWAS) with the aim of detecting QTL affecting the disease traits, testing one SNP at a time. The assumed model was

y=Xb+Zu+wα+e,

where **y**, **X**, **b**, **Z,** and **u** are the same as in GBLUP, **w** is the vector containing the SNP allelic dosage for each fish in **y** and α is the SNP substitution effect that was included as a fixed effect.

In order to estimate the proportion of the total genetic variance explained by putative candidate regions identified through the GWAS, a regional heritability analysis (RHA) assuming the same mixed linear model was performed. For that, a reduced genomic relationship matrix constructed from the SNPs covering a specific putative QTL region previously identified by GWAS was used instead of the **G** matrix computed with all SNPs. Both GWAS and RHA were performed using the software DISSECT (Canela-Xandri et al., 2015). Significance of association in GWAS was assessed using a false discovery rate (FDR) multi-test correction threshold at the 5% level following the Benjamini–Hochberg procedure (Benjamini and Hochberg, 1995). Adjusted *p*-values were obtained with the software Myriads (Carvajal-Rodríguez, 2018).

Regions flanking SNPs significantly associated with the traits in the GWAS were explored for gene content using the turbot genome browser^1^ (Figueras et al., 2016). These regions comprised ±500 kb up and downstream from a particular candidate SNP. The genes identified were further analysed using BLAST2GO (Conesa et al., 2005) to determine whether their specific functions were significantly enriched when compared to those of all genes mapped in the turbot transcriptome.

**Table 2:** Number of data (*N*) and average number of days (standard deviation) for the different traits.

		Uncensored data		Censored data	
Trait	Trial	*N*	Average (SD)	*N*	Average (SD)
Resilience	1	470	52.10 (18.46)	209	104.00 (0.00)
	2	457	110.33 (27.55)	250	160.00 (0.00)
Resistance	1	412	42.74 (17.86)	171	94.95 (22.76)
	2	344	101.45 (28.51)	238	158.76 (12.24)
Endurance	1	375	9.54 (8.75)	37	50.89 (28.12)
	2	339	9.81 (9.64)	5	60.40 (67.83)

*Resilience was measured as the number of days until death; resistance, as the number of days to the onset of visual signs, and endurance, as the number of days from the onset of visual signs to death.*

### Data Availability

The data that support the findings of this study are available from CETGA (Cluster de Acuicultura de Galicia), but restrictions apply to the availability of these data, which were used under licence for the current study, and so are not publicly available. Data are however available from the corresponding author upon reasonable request and with permission of CETGA.

## Results

Table 2 gives a description of the data available for the three traits in both trials. For instance in trial 1, 470 fish died during the experiment (uncensored data for resilience) and 209 survived (censored for resilience). From these 679 initial fish, 412 showed visual signs (uncensored for resistance) and 171 survived without showing any visual sign (censored for resistance). Fish that died without showing visual signs are not considered for resistance but they are included in the uncensored group for resilience. From the 412 fish that showed visual signs, 375 died (uncensored for endurance), and 37 survived (censored for endurance).

### Phenotypic (Co)variation

**FIGURE 1:**
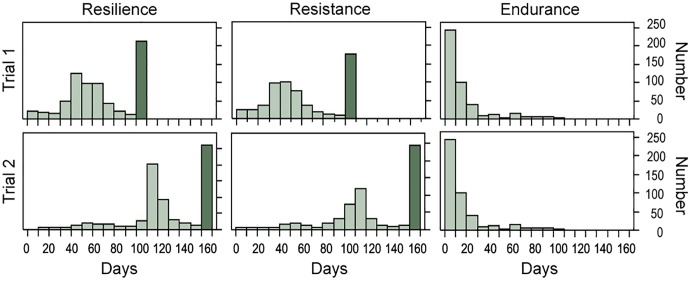
Number of uncensored (light) and censored (dark) data in successive 10-day periods for resilience, resistance, and endurance, and both trials.

**FIGURE 2:**
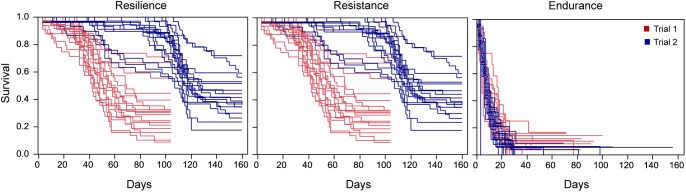
Kaplan–Meier survival curves for trials 1 and 2 when fish are grouped by recipient families for resilience, resistance, and endurance.

Phenotypic distributions for resilience, resistance and endurance for the two trials are presented in Figure 1. Within trial, distributions of time to death (resilience) and time to onset of visual signs (resistance) were very similar, while time from the onset of visual signs to death (endurance) showed a different distribution pattern. Endurance showed similar average survival times in trials 1 and 2, while resilience and resistance showed values about double in trial 2 than in trial 1 for uncensored data (Table 2). This large variation observed between trials could be due to a lower virulence of the pathogen isolate used in trial 2 given that a later onset of visual signs in recipient fish were found in this trial (Figure 2). In fact, trial 2 lasted 56 days more than trial 1. However, Anacleto et al. (2019) showed that variation in time to the appearance of visual signs and in time from signs to death was similar in both trials. This suggests that despite the different virulence of the pathogen strain, the same traits were recorded in both trials.

The differences between trials justified the stratification of the baseline hazard by trial for the three traits in the survival analysis, particularly for resilience and resistance. Kaplan–Meier survival curves (Figure 2) provided evidence of phenotypic variation across recipient families for the three traits, suggesting an underlying genetic component for these traits.

The hypothesis of a Weibull distribution was graphically tested (Supplementary Figure 1). Although *R*^2^ from the linear regression analysis was high (>0.84), the graphs showed deviations from linearity. This result justifies the use of both Cox and Weibull models for the estimation of genetic parameters. The estimates of the Weibull parameters (i.e., the slope or shape parameter ρ and the intercept or ρ log λ, where λ is the scale parameter) for resilience, resistance and endurance for trial 1 (trial 2) were, respectively, 2.23 (4.77) and -9.12 (-23.37), 1.95 (2.90) and -7.65 (-14.28), and 1.23 (1.61) and -3.04 (-3.46). The fact that slopes were >1 implies that the hazards increased overtime, as expected. The different estimates of ρ and λ obtained for both trials again justified the fit of different baseline hazards for each trial.

### Environmental Effects

The values of Maddala’s R_M_^2^ indicated that the proportion of variation explained by the model for the three traits decreased by about 5% when the initial weight was excluded from the model. This proportion decreased up to 15% for endurance and to 35% for both resilience and resistance when tank was the effect excluded. Differences in the relative risk of death or of showing visual signs across weight categories were not significantly different, although a general trend was observed. The larger the initial weight the lower was the risk (Supplementary Figure 2). In particular, the category of fish with the lowest weight (<25.0 g) had a 1.5-fold higher risk of death (resilience and endurance) than the category with the highest weight (>115.3 g). No apparent association between the initial weight and the onset of visual signs (resistance) was detected. Different risks of death and risks of showing visual signs were also observed for fish maintained in the different tanks. A maximum difference of ∼36-fold higher risk of death or showing signs was observed between tanks. In particular, 27 tanks showed significant risks of death and 14 tanks showed significant risk of showing signs. Only one tank presented a significantly higher risk of death due to infection (∼ninefold) than other tanks (Supplementary Figure 2).

**Table 3:** Estimates of additive genetic variance (${\text{σ}}_{\text{u}}^{2}$) and heritability in the logarithmic ($$h_{\log}^{2}$$), and original scale (*h*^2^) derived from survival analyses under Cox and Weibull models

Trait	Model	*N*	${\text{σ}}_{\text{u}}^{2}$	$$h_{\log}^{2}$$	*h*^2^
Resilience	Cox	1,386	0.258	0.135	0.147
	Weibull		0.399	0.195	0.211
Resistance	Cox	1,165	0.561	0.254	0.255
	Weibull		0.618	0.273	0.274
Endurance	Cox	756	0.142	0.079	0.118
	Weibull		1.927	0.742	0.644

*Corresponding number of data (N) available for each analysis is also indicated.*

**Table 4:** Genetic (above diagonal) and phenotypic (below diagonal) correlations between disease resistance traits and between disease resistance traits and growth derived from linear model analyses.

	Resilience	Resistance	Endurance	Growth
Resilience		0.904 (0.845, 0.941)	0.765 (0.429, 0.915)	0.669 (0.362, 0.845)
Resistance	0.967 (0.962, 0.971)		0.358 (−0.566, 0.884)	0.697 (0.277, 0.893)
Endurance	0.084 (0.010, 0.156)	−0.152 (−0.223, −0.080)		0.179 (−0.554, 0.756)
Growth	0.228 (0.166, 0.288)	0.231 (0.161, 0.300)	0.007 (−0.067, 0.080)	

*Ninety-five percent confidence intervals are indicated in brackets.*

### Genetic Co-variation

**FIGURE 3:**
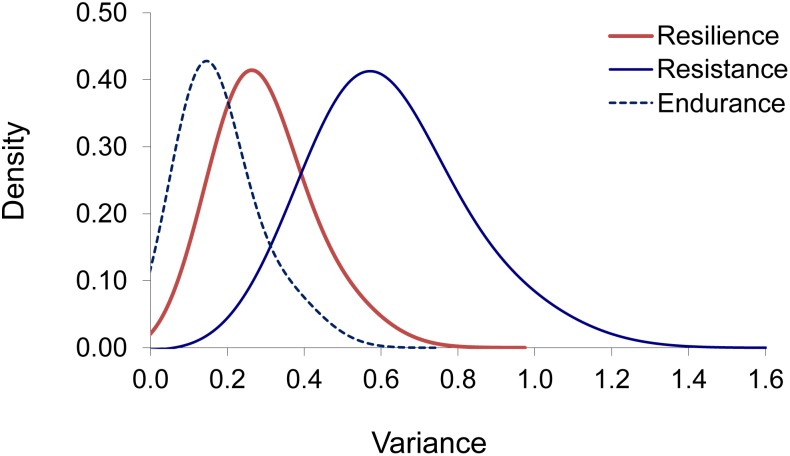
Gram–Charlier approximations of the posterior density distributions for the additive genetic variance under Cox models for resilience, resistance, and endurance.

Estimates of additive genetic variances and heritabilities derived from survival analyses are given in Table 3. Estimates of heritability when assuming the Cox model were slightly lower than those obtained when assuming the Weibull model for resilience (0.15 vs. 0.21) and resistance (0.26 vs. 0.27). For endurance, however, the heritability estimated when assuming the Weibull model was unexpectedly much higher (0.64) than that obtained when assuming the Cox model (0.12). Gram–Charlier approximations (Figure 3) showed that the posterior distributions for the additive genetic variance were significantly different from zero (probability > 0.95) when assuming the Cox model.

The genetic correlation between resilience and resistance was very high (0.90) indicating that both are, at a large extent, the same trait. The genetic correlation between resilience and endurance, although lower, was also high (0.77) and much higher than the phenotypic correlation (Table 4). It is worth mentioning that although the phenotypic correlation between resistance and endurance was negative, the genetic correlation between both traits was not significantly different from zero. These results were in accordance with those obtained from Pearson correlations between estimated breeding values obtained from survival analysis assuming Cox animal models (Supplementary Figure 3). The genetic correlation between growth and endurance was not significantly different from zero (Table 4) but those between growth and resilience, and growth and resistance were high and positive (0.67 and 0.70, respectively).

### Accuracy of Genomic Selection and Detection of QTL Regions Associated With the Traits Defining the Host Response to Infection

**FIGURE 4:**
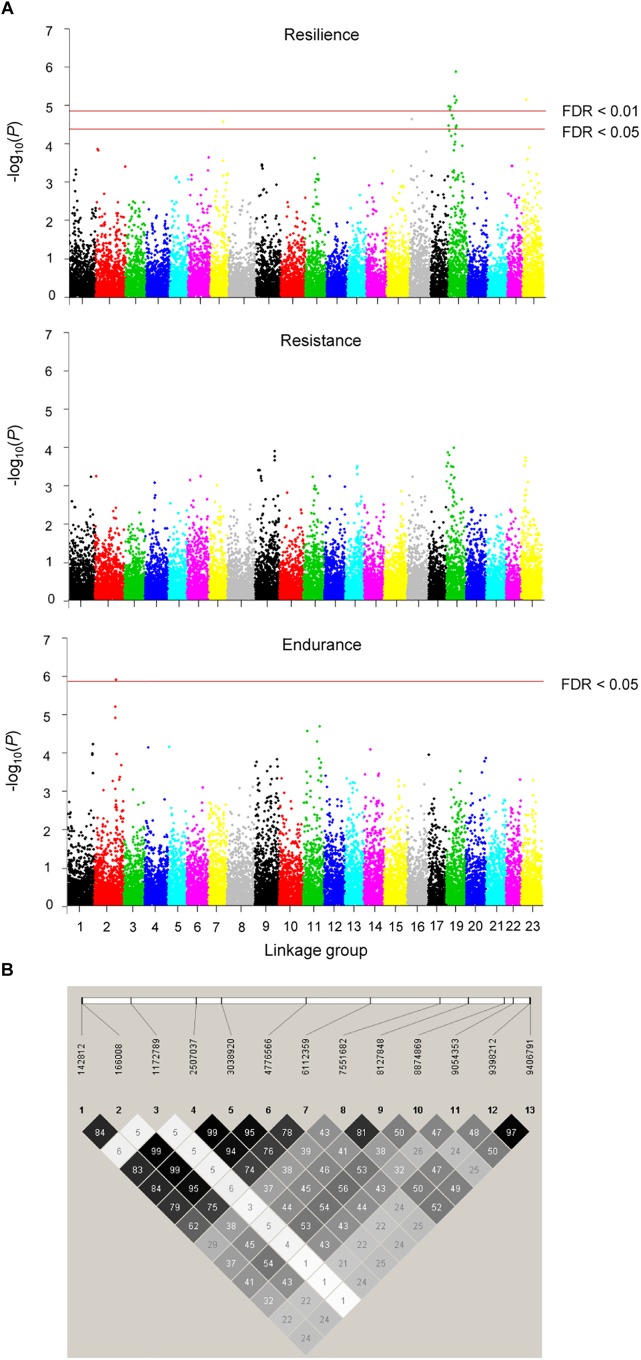
Manhattan plots resulting from the GWAS for resilience, resistance, and endurance at two different false discovery rate thresholds (FDR = 1 or 5%) **(A),** and linkage disequilibrium (*r*^2^) plot for the 13 significant SNPs identified in the 9.3 Mb candidate QTL region for resilience **(B)**. Colour intensity of diamonds is proportional to *r*^2^ values, which are given in percentage.

The accuracy of genomic breeding values for resilience, based on the 10-fold cross-validation results, was 12% higher than that of pedigree-based breeding values (0.46 vs. 0.41, respectively). GWAS revealed 16 significant SNPs associated with resilience (FDR < 0.05). Thirteen of them were located on LG19 and the remaining three were located on LG7, LG16, and LG23 (Figure 4A). Twelve of the SNPs on LG19 were part of the same linkage block (Figure 4B) that presented an average linkage disequilibrium, measured as the squared correlation between pairs of loci (*r^2^*) of 0.51, and showed an average effect on resilience of 4.66 (SD = 0.42) days. Their minor allele frequencies ranged from 0.15 to 0.39 (Table 5). The region delimited by the 13 SNPs spanned 9.3 Mb (from 142,812 to 9,406,791 bp), included 98 SNPs and explained 33% of the total genetic variance for the trait according to the RHA analysis. No significant associations were found for resistance, although the pattern of -log(*p*) values was very similar to that obtained for resilience. For endurance, one significant association was detected on LG2 (Figure 4A).

Functional enrichment of the candidate region at LG19 using the turbot transcriptome as a reference revealed 32 genes with functions related to the immune or defence system. These functions were associated with (i) tissue regeneration; (ii) response to wounding; (iii) leukotriene production involved in inflammatory response (Funk, 2001); (iv) activation of NF-kappaB-inducing kinase activity involved in the regulation of the immune system (Thu and Richmond, 2010); (v) toll-like receptor 21 signalling pathway involved in the innate immune response (Akira and Takeda, 2004); (vi) activation of MAPK activity involved in innate immune response (Vidal et al., 2001); and (vii) cellular defence response. Seven of these genes had been previously suggested to be involved in the response to infectious diseases in turbot (Supplementary Table 1).

## Discussion

Quantifying the host genetic contribution to epidemic risk and severity remains a long-standing challenge in infectious disease research. In this study, survival analysis and linear models were used to disentangle the genetic basis of resistance and endurance to disease, using scuticociliatosis in turbot as a model and taking benefit from both pedigree and genomic information. The results showed the existence of genetic variation for both traits and also for resilience, the composite trait that includes both resistance and endurance. Estimates of heritability were 0.26, 0.12, and 0.15 for resistance, endurance and resilience, respectively. The genetic correlation between resilience and resistance and that between resilience and endurance were high. However, there was no evidence of the existence of a genetic correlation between resistance and endurance.

To the best of our knowledge, only few studies have attempted to formally decompose genetic variation in survival in fish populations (Ødegård et al., 2011b; Mazé-Guilmo et al., 2014). Using a cure survival model, Ødegård et al. (2011b) decomposed the observed time to death after an infection challenge into two components, susceptibility (ability to avoid infection) and endurance, from records on daily survival in Pacific white shrimp challenged with the Taura syndrome virus. This type of approach attempts to distinguish susceptible from non-susceptible (“cured”) survivors. In contrast, in our study, we used the appearance of visual signs as a proxy for time of onset of disease and thus individual phenotypes for resistance and endurance were obtained in an easy and non-invasive way (Anacleto et al., 2019). In line with our findings, Ødegård et al. (2011b) found that the heritability for the ability of fish to avoid developing infection was larger than for endurance (0.41 and 0.07, respectively).

In this study, we distinguish resistance and endurance as the two different genetic host traits affecting host survival to infectious challenge. A much more common approach is to distinguish between host resistance and tolerance as the two alternative host defence mechanisms to infection, where resistance refers to the propensity of a host to prevent infection or limit its extent, and tolerance refers to the propensity of the host to reduce fitness loss (e.g., death) under infection (Simms and Triplett, 1994; Roy and Kirchner, 2000; Restif and Koella, 2004; Detilleux, 2012). Based on these definitions, endurance and tolerance are clearly closely related. However, operationally, tolerance is typically defined as the slope of a regression of host fitness against pathogen burden (Simms, 2000; Råberg et al., 2007; Kause, 2011; Medzhitov et al., 2012). Estimating tolerance slopes, thus requires measures of pathogen burden. However, obtaining informative measures of pathogen burden is a costly and often difficult task because animals are infected at different time points and the pathogen burden changes during the course of infection (Ayres and Schneider, 2012; Doeschl-Wilson et al., 2012). The benefit of using endurance over tolerance is that it does not require measures of pathogen burden and that endurance estimates are not hampered by the statistical constraints of reaction norms (Kause and Ødegård, 2012; Lough et al., 2017). However, it also prevents direct comparison between our endurance estimates with tolerance estimates reported in the literature, despite the fact both traits are closely related.

Estimates of heritability tended to be higher under the Weibull than under the Cox model, especially for endurance (0.64 vs. 0.12, respectively). The fact that the graphical test of the Weibull assumption (Supplementary Figure 1) showed deviations from the linear pattern of response expected under this model might partially explain the differences in the estimates. Estimates of genetic variance under sire models were also obtained to substantiate those obtained under animal models. Conceptually, animal models are preferred over sire models because they take into account both male and female paths of gene transmission and because genetic variability is better assessed from the variability in genetic values of individuals than from variability among a reduced number of sires. This is especially critical when sample size is not large, as in our case. However, sire models could have some advantage to control bias associated with confounding between genetic and contemporary group effects, especially when the amount of uncensored data in contemporary group is small (Jenko et al., 2013). In aquaculture settings, the contemporary group is represented by the tank effect, which includes the effect of the environmental conditions of the tank (temperature, water quality, etc.) as well as the level of infectious challenge produced by the infected tank members. Estimated heritabilities from sire models for endurance under Cox and Weibull models were closer to each other (0.07 and 0.13, respectively) than under animal models, supporting the heritability estimates obtained assuming Cox in the animal model. In addition, linear animal models were also explored for the three disease traits (data not shown). Heritability estimates under linear animal models were substantially lower than the estimates obtained using survival analysis for the three traits, which also questions the high genetic variation estimated with the Weibull animal model for endurance. However, linear models cannot accommodate the use of censored information or the change of the effect of factors over time, which implies a suboptimal use of the available information. Overall, in our case, survival analysis under a Cox animal model seems to be the method of choice, since it avoids the assumption of the data following a Weibull distribution, enables the use of all available information (censored and uncensored data) and provides a flexible modelling framework, allowing to accommodate differences in the two trials in the baseline behaviour of the traits along the time of the experiment.

**Table 5:** Physical position (Pos), minimum allele frequency (MAF), estimated allele substitution effect (in days of survival), standard error (SE), and *p*-value for the significant SNPs identified in LG19.

Pos (bp)	MAF	Effect	SE	*p*-value
142,812	0.18	4.81	0.583	1.01E-05
166,008	0.16	4.78	0.591	3.29E-05
1,172,789	0.22	−4.20	0.603	4.43E-05
2,507,037	0.15	5.12	0.595	1.23E-05
3,038,920	0.16	5.07	0.593	1.03E-05
4,776,566	0.16	4.99	0.592	1.73E-05
6,112,359	0.18	4.85	0.621	2.15E-05
7,551,682	0.28	4.45	0.619	5.62E-06
8,127,848	0.26	4.45	0.619	8.14E-06
8,874,869	0.20	5.10	0.598	1.25E-06
9,054,353	0.25	4.33	0.640	3.61E-05
9,398,212	0.39	4.16	0.638	6.91E-06
9,406,791	0.38	3.82	0.631	3.27E-05

Our estimates of both phenotypic and genetic variances were larger for resistance than for endurance, in agreement with previous studies in fish (Ødegård et al., 2011b). This may indicate that fish vary genetically more in whether and when they develop disease than in how they cope with the infection. This is useful from an epidemiological point of view as fish that are less likely to become infected or diseased are less likely to infect others. However, the estimated heritability for endurance in our study may be biased. This could be, at least in part, a consequence of how traits were measured. In particular, visual signs may indicate a progressed disease state rather than time of onset of infection, and this may explain why there is more genetic and phenotypic variance in resistance than in endurance. Also, there may be additional variation between time of onset of infection and time when visual signs develop. That is now attributed to resistance but may refer to a coping mechanism and hence could be arguably attributed to endurance. An indication of this may be that some infected individuals after post mortem examination did not develop visual signs in trial 2 (Anacleto et al., 2019). This may partly explain why there is more genetic and phenotypic variance in resistance than in endurance.

The availability of genomic information obtained from RAD-sequencing for 36 families allowed us to identify a candidate QTL region for resilience on LG19 that explains 33% of the additive genetic variance. A preliminary study to identify QTLs for survival to *P*. *dicentrarchi* was addressed by Rodríguez-Ramilo et al. (2013). Using a linkage mapping approach with four families and 98 microsatellite markers, they detected a significant QTL for resilience that explained up to 22% of the phenotypic variance and that was located on LG3. The discrepancy with our results may be due to different reasons. Here, we have carried out a more in depth analysis of the turbot response to *P*. *dicentrarchi* by using high-throughput SNP genotyping and a more refined map, and have analysed a larger amount of families. Also, both studies differ in the via of infection used in the challenge tests (injection vs. cohabitation) and thus the traits analysed may not be necessarily the same. Although the GWAS for resistance had not enough power to detect SNPs significantly associated to this trait (the number of data available was lower for resistance than for resilience), the pattern of -log(*p*) values in the Manhattan plot was very similar to that for resilience, supporting the hypothesis that both traits are, to a large extent, the same. The pattern found for endurance was however different, which may suggest that this trait is controlled by different genes.

Mining on the QTL region revealed several genes related with immune response and defence mechanisms, some of them previously identified for turbot infectious diseases, including infections with *P. dicentrarchi* (Pardo et al., 2012), viral hemorrhagic septicemia virus (Pereiro et al., 2016) and *Aeromonas salmonicida* (Millán et al., 2011). Interesting genes include the *DMBT1* and *MARCH8* genes (involved in the activation of innate immune and defence response, and the adaptive immune response), the *PLEK* gene (involved in the regulation of response to stress) and the *TRIM16* gene (involved in the regulation of host antiviral activities mediated by cytokines). Validation of the candidate region identified here in other genetic backgrounds is required, but it may be an appropriate target for MAS, and for functional studies designed to investigate the underlying causative genes. An alternative approach for improving disease traits would be to perform genomic selection (Meuwissen et al., 2001). Here, the accuracy of genomic prediction of breeding values outperformed by 12% the accuracy of pedigree-based prediction. This is in line with the comparative performance observed in gilthead seabream (Palaiokostas et al., 2016), European seabass (Palaiokostas et al., 2018a), and common carp (Palaiokostas et al., 2018b). Improvement in prediction accuracy for these species was 27–53, 13, and 18%, respectively.

In contrast with other aquaculture species such as white shrimp (Argue et al., 2002), rainbow trout (Henryon et al., 2003), and coho salmon (Yáñez et al., 2016) where negative genetic correlations between growth and resistance to disease have been reported (Taura syndrome virus, viral haemorrhagic septicaemia and salmon rickettsial syndrome, respectively), we found that resilience and resistance were favourably genetically associated with growth. This might have important implications in selective breeding programmes, since no detrimental effects on resistance to scuticociliatosis are expected from current selection on growth, which is the main breeding objective in turbot breeding programmes. However, this result should be interpreted with caution given that here we considered growth in experimental challenged fish, which may not represent growth in commercial settings.

In the context of animal breeding, understanding whether resistance and endurance vary independently or are antagonistically related (i.e., they represent alternative, mutually exclusive defence mechanisms) is fundamental in order to determine optimal disease control strategies. Here, genetic correlations between resilience and its two components (resistance and endurance) were positive and high, indicating that selection on resilience will lead to improvement in both defence mechanisms. However, when dealing with infectious diseases, ignoring the impact of selection on disease transmission (i.e., ignoring infectivity) can lead to undesired outcomes. Individuals with greater endurance to infection will live longer when infected and therefore may have more chances of infecting others. The negative side-effects are exacerbated if the hypothesis that tolerance and hence also endurance mechanisms play a role in the maintenance of the asymptomatic superspreader state is true (Gopinath et al., 2014), as selection for resilience or tolerance (and hence endurance) would maintain infection in the population. From an epidemiological viewpoint, selection for resistance would therefore be a better option (Doeschl-Wilson et al., 2011). In agreement with the results from previous fish studies disentangling resistance and endurance (Ødegård et al., 2011b), the genetic correlation between both traits was not significantly different from zero. However, selecting for resistance against developing disease would require a measure of the time of onset of visual signs (which may not be feasible in large scale practical aquaculture breeding programmes) or using non-standard statistical models such as cured models (Kause and Ødegård, 2012). In any case, genomic selection would be an alternative strategy as recording phenotypes could be substantially reduced since estimates of SNP effects can be used across generations. Anyhow, there is a need to understand to what extent these conclusions apply to other diseases, and how they are affected by the trait definition and measures.

## Ethics Statement

This study was carried out in accordance with the recommendations of the ethical regulations and with the approvement of the Regional Government of Xunta de Galicia (registered under the code ES150730055401/ 16/PROD.VET.047ROD.01).

## Author Contributions

AD-W, OA, SC, and BV contributed to the conception and design of the study. SC carried out the experiments. MS, MC, and AF performed the statistical analyses. FM, AM, MH, CF, and PM developed the genomic tools and carried out the data mining. MS and BV wrote the first draft of the manuscript. All authors contributed to the discussion of results and the edition of the manuscript revision.

## Conflict of Interest Statement

The authors declare that the research was conducted in the absence of any commercial or financial relationships that could be construed as a potential conflict of interest. The reviewer CP declared a past co-authorship with one of the authors PM to the handling editor.

## Footnotes

1 http://denovo.cnag.cat/genomes/turbot/
