# Supplementary material for: Disentangling Genetic Variation for Resistance and Endurance to Scuticociliatosis in Turbot Using Pedigree and Genomic Information
Source: Front Genet. 2019 Jun 7;10:539. doi: 10.3389/fgene.2019.00539 (PMC6565924; doi:10.3389/fgene.2019.00539)

Supplementary Material

Disentangling genetic variation for resistance and endurance to scuticociliatosis in turbot using pedigree and genomic information

María Saura[1*], María J. Carabaño[1], Almudena Fernández[1], Santiago Cabaleiro[2], Andrea B. Doeschl-Wilson[3], Osvaldo Anacleto[3], Francesco Maroso[4], Adrián Millán[4], Miguel Hermida[5], Carlos Fernández[5], Paulino Martínez[5] and Beatriz Villanueva[1]

*** Correspondence:** María Saura, saura.maria@inia.es

Supplementary Figure 1 Graphical test for the Weibull model depicting log (−log *S*(*t*)) against log (*t*) and adjusted linear trends for resilience (A), resistance (B) and endurance (C) for the survival function S(*t*), estimated by the Kaplan–Meier method.

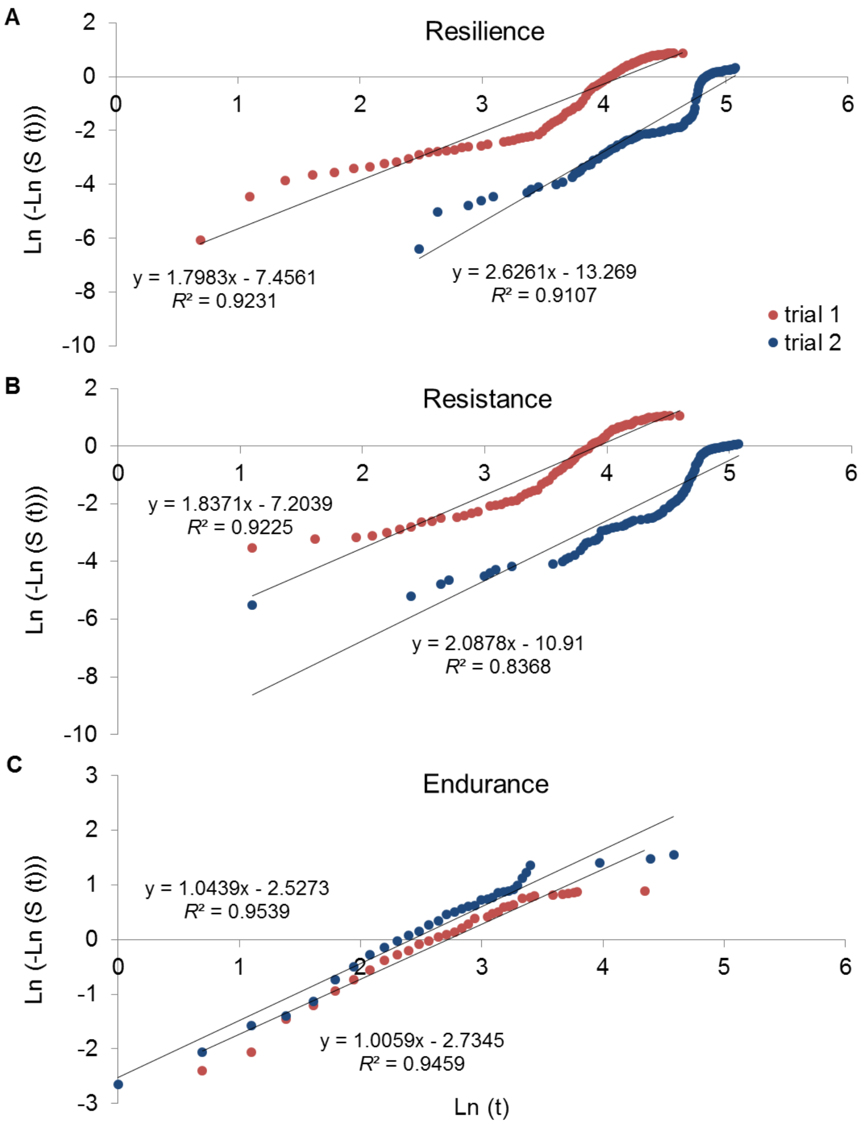

Supplementary Figure 2 Relative risks associated to initial weight (A) and tank (B) effects for resilience, resistance and endurance. Ten classes were considered for initial weight (≤ 24.9, 25.0−29.6, 29.7−34.6, 34.7−40.6, 40.7−49.5, 49.6−65.3, 65.4−81.3, 81.4−94.8, 94.9−115.2, 115.3−196.3) and 71 classes for tank.

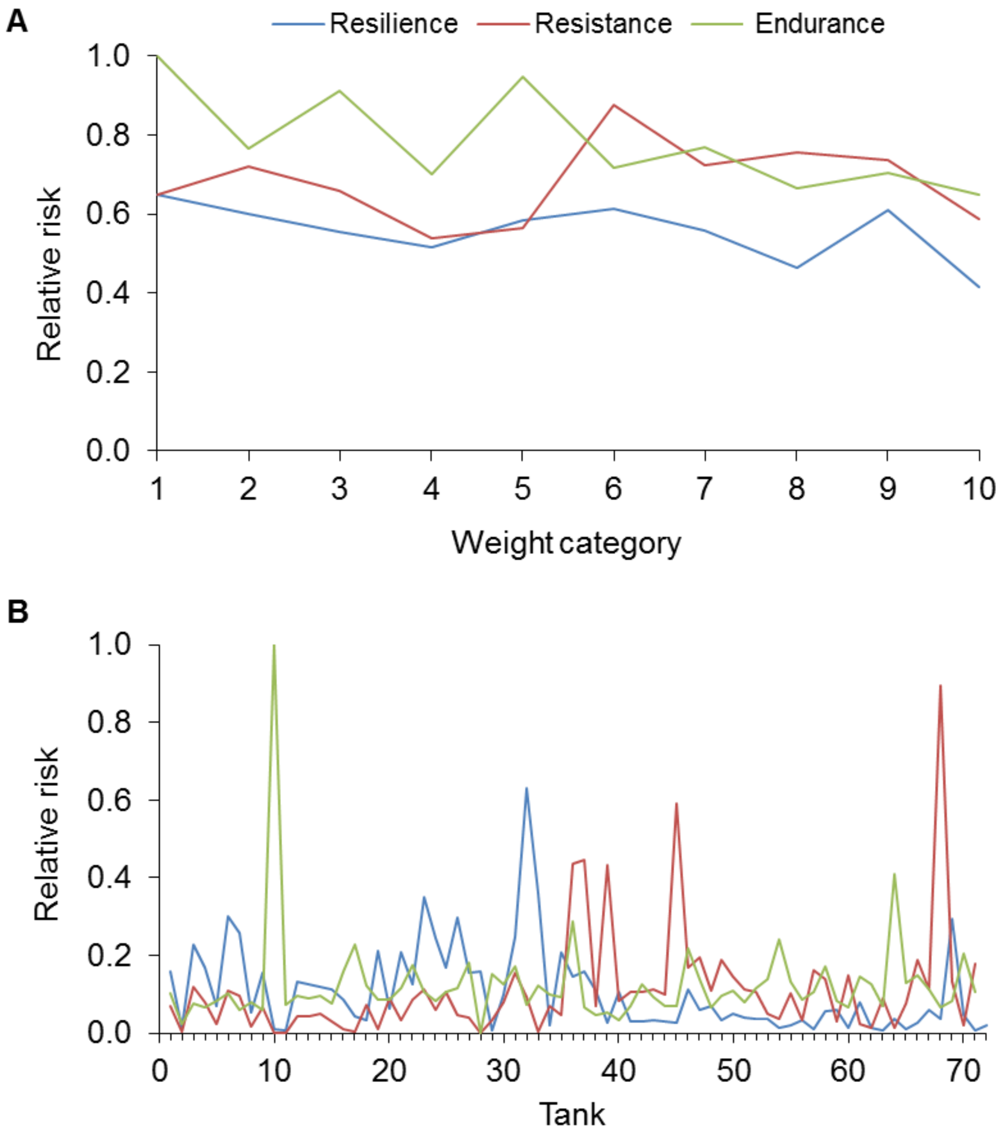

Supplementary Figure 3 Regression of estimated breeding values (EBV) obtained from survival analysis assuming Cox animal models for resilience, resistance and endurance.

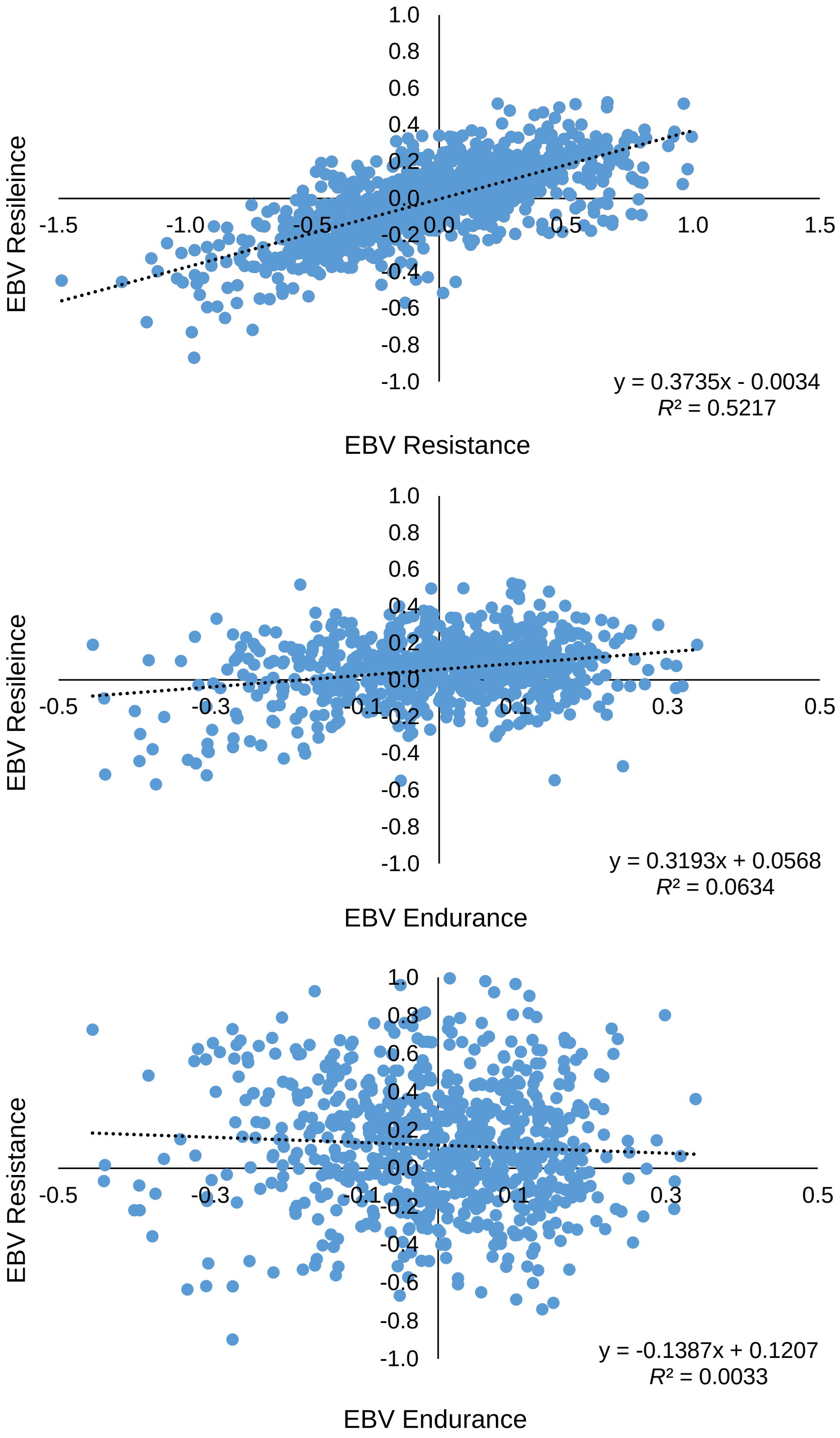

Supplement: Supplementary file 1 [file Data_Sheet_1.PDF]
